# Undetected Multidrug-Resistant Tuberculosis Amplified by First-line Therapy in Mixed Infection

**DOI:** 10.3201/eid1907.130313

**Published:** 2013-07

**Authors:** Suzanne M. Hingley-Wilson, Rosalyn Casey, David Connell, Samuel Bremang, Jason T. Evans, Peter M. Hawkey, Grace E. Smith, Annette Jepson, Stuart Philip, Onn Min Kon, Ajit Lalvani

**Affiliations:** Imperial College London, London, UK (S.M. Hingley-Wilson, R. Casey, D. Connell, S. Bremang, A. Lalvani);; Heart of England National Health Service Foundation Trust, Birmingham, UK (J.T. Evans, P.M. Hawkey, G.E. Smith);; School of Infection and Immunity, University of Birmingham, Birmingham (P.M. Hawkey);; Imperial College National Health Service HealthcareTrust, London (A. Jepson, S. Philip, O.M. Kon); 1 Current affiliation: University of Surrey, Guildford, UK.

**Keywords:** Mycobacterium tuberculosis, co-infection, treatment resistance, multidrug resistant, drug sensitive, tuberculosis and other mycobacteria, antimicrobial resistance, bacteria, TB

## Abstract

Infections with >1 *Mycobacterium tuberculosis* strain(s) are underrecognized. We show, in vitro and in vivo, how first-line treatment conferred a competitive growth advantage to amplify a multidrug-resistant *M. tuberculosis* strain in a patient with mixed infection. Diagnostic techniques that identify mixed tubercle bacilli populations are needed to curb the spread of multidrug resistance.

As the number of multidrug-resistant tuberculosis (TB) cases continues to rise, so does the amplification of multidrug-resistant *Mycobacterium tuberculosis* strains during treatment (*1*,*2*). This amplification is generally assumed to result from in vivo evolution of drug resistance caused by poor therapy compliance or, in high-incidence settings, from exogenous reinfection with a multidrug-resistant strain. We report a case in which emergence of multidrug resistance did not result from in vivo acquisition of drug resistance by a drug-sensitive strain or from exogenous reinfection with an already resistant strain. By integrating epidemiologic, microbiological, and molecular strain typing data with in vitro competitive growth experiments, we provide evidence for an initial mixed infection with a drug-sensitive strain and an undetected drug-resistant strain that outgrew the sensitive strain under the selection pressure of first-line chemotherapy.

*M. tuberculosis* strains in sputum from TB-infected patients or in samples from the disease site are generally identified by strain typing a single broth culture or colony grown on solid medium. However, this method does not enable identification of mixed infections, and any treatment regimen would be determined on the basis of the drug sensitivity of the strain with the fastest growth rate in the in vitro culture. Use of suboptimal drug combinations could lead to selection of a slower growing, drug-resistant strain already present in the host and thus to treatment failure.

Studies of artificially mixed *M. tuberculosis* strains before and after culture showed that culturing can reduce the clonal complexity of the strains and that, in most samples (6/10), only 1 strain will be identified in mixed infections after culture (*3*). This suggests that mixed infections and clonal complexity are underrepresented in culture-based diagnoses of TB. In support of this suggestion, the results of molecular-based methods that use strain-specific PCR showed that 2.1%–19.0% of patients with active TB in moderate to high incidence countries were simultaneously infected with >2 strains (*1*,*2*,*4*–*10*).

Possible co-infection of patients with drug-sensitive and drug-resistant *M. tuberculosis* strains has been described (*1*,*2*), and modeling of the effect of such co-infection on the long-term dynamics of tuberculous infection has led to the hypothesis that persons with this type of infection may retain small populations of drug-resistant bacteria that can flourish after the host receives treatment (*11*). van Rie et al. showed the amplification of a drug-resistant strain after treatment and postulated selection of drug-resistant strains from an initial mixed infection through antimicrobial drug pressure (*2*). We confirm this hypothesis by combining detailed longitudinal clinical and microbiological observation with the use of novel in vitro growth competition assays to study 2 co-infecting patient strains in the presence and absence of the primary drug used in treatment. 

## The Study

The 2 *M. tuberculosis* strains were isolated from a 68-year-old man from Portugal. He did not have HIV and was treated as a confined inpatient, limiting the possibility that this was not a true in vivo mixed infection. Using a novel approach, we correlated in vitro growth and treatment characteristics for the patient strains with the in vivo strain predominance and persistence of a less-fit, drug-resistant strain. All samples were obtained with approval from St. Mary Hospital’s (London, UK) Research Ethics Center (no. 07/H0712/85) and with the patient’s written informed consent.

Details of the patient samples are in the Table. The initial bronchoalveolar lavage smear sample was positive for acid-fast bacilli (AFB); culture results were positive for fully sensitive *M. tuberculosis*. Treatment with isoniazid, rifampin, ethambutol, and pyrazinamide was begun. Because of the patient’s alcohol use, his treatment was managed on an inpatient basis in a single-patient, negative-pressure room. Two months later, repeat sputum smears were positive for AFB, and culture results were positive for fully sensitive *M. tuberculosis*. After 4 months of treatment, the patient’s clinical signs had not improved, and his sputum smear was still positive for AFB. Culture results for the sputum sample were positive for *M. tuberculosis* resistant to isoniazid and ethambutol; a modified treatment regime resolved the infection, and the patient was released the following month, by which time his smear and culture results were negative.

**Figure 1:**
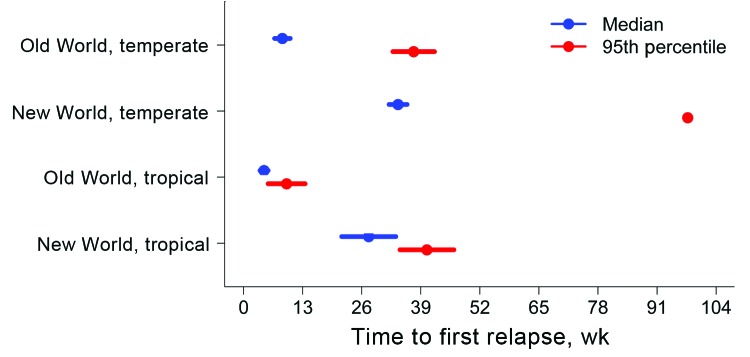
*Mycobacterium tuberculosis* co-culture competition experiment in a study of the amplification of multidrug resistance induced by first-line treatment of a mixed *M. tuberculosis* infection. The results suggest competitive advantages in vitro, which may account for patient strain phenotype in vivo. S, drug sensitive; R, drug resistant; OADC, oleic acid, albumin, dextrose, catalase growth supplement; OD_600_, optical density read at 600 nm; −INH, without isoniazid; +INH, with INH; MIRU-VNTR, mycobacterial interspersed repetitive unit–variable number tandem repeat; CFU, colony-forming units.

We molecularly characterized the strains by using mycobacterial interspersed repetitive unit–variable number tandem repeat (MIRU-VNTR) typing (*12*); results showed that the drug-sensitive and drug-resistant *M. tuberculosis* strains were 2 distinct strains (Table) rather than 1 sensitive strain that had become resistant through mutagenesis. Because the patient was isolated while an inpatient, exogenous reinfection with a primary drug-resistant strain was ruled out. In addition, treatment compliance was directly observed, so in vivo development of drug resistance caused by poor compliance was also ruled out. Thus, it is highly likely that the patient was harboring a mixed infection of drug-sensitive and drug-resistant strains when he initially sought care at the clinic. Such a co-infection would not have been detected because single-colony or broth cultures are commonly used for strain typing, and these techniques would give the fastest growing strain a competitive advantage. Thus, we devised a competitive growth assay to determine if the patient had a mixed infection and to provide correlating in vitro and in vivo evidence of mixed infection (Figure 1).

**Figure 2:**
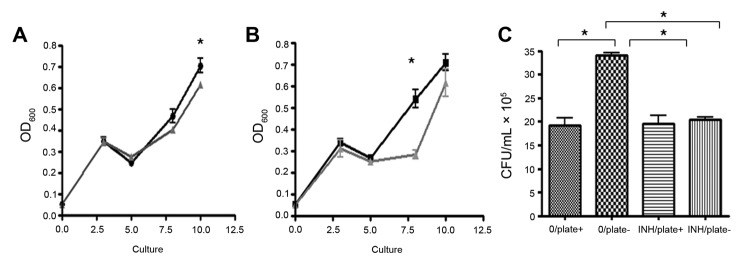
Analyses of the amplification of multidrug-resistant *Mycobacterium tuberculosis* during treatment of a drug-sensitive (S) strain in a mixed infection (i.e., infection with drug-resistant [R] and S strains). In the presence of isoniazid (INH), the faster growing S strain lost its competitive advantage, and the R strain became more prevalent. A–C) Data are means of 3 independent replicates with SE bars. A) Single strain growth analyses of S (black circles) and R (gray triangles) *M. tuberculosis* strains. Growth was measured by optical density at 600 nm (OD_600_). *p<0.05. B) Competitive growth analyses of mixed strains alone (black squares) and with 0.2 μg/mL INH (gray triangles). Growth, in triplicate, in 7H9 broth plus OADC (oleic acid, albumin, dextrose, and catalase growth supplement), glycerol, and Tween 80 was measured by optical density at OD_600_. Statistical analyses were performed on triplicate samples by using 2-way analysis of variance. *p<0.05. C) Identification of the predominant strain in mixed cultures with and without 0.2 μg/mL INH (INH/plate+ and INH/plate−, respectively). Strains were identified on day 7 by plating a 10-fold dilution series of co-cultures onto 7H10 agar, plus OADC and glycerol, with or without 0.2 μg/mL INH (0/plate+ and 0/plate−, respectively) and incubating for 2 weeks at 37°C. Statistical analyses were conducted on triplicate samples by using a 2-tailed *t*-test. *p<0.02. CFU, colony-forming units.

For the in vitro growth analyses of the 2 strains, we inoculated broth cultures and measured growth at an optical density of 600 nm, characterized the dominant strain by using MIRU-VNTR, and quantified colony-forming units on agar plates in the presence and absence of isoniazid. At several points during logarithmic growth, the drug-sensitive strain grew substantially faster than the resistant strain (Figure 2, panel A), suggesting that without the selective pressure of isoniazid, the sensitive strain would be most prevalent in a mixed infection. 

For the in vitro competition assays, the strains were mixed (1:1), and isoniazid (0.2 μg/mL) was or was not added before measurement of growth and determination of the dominant strain. In the presence of isoniazid, the growth rate was lower, suggesting that the drug-resistant strain outcompeted the drug-sensitive strain to become the dominant strain (Figure 2, panel B). This was confirmed by MIRU-VNTR typing and growth analyses (Figure 2, panel C). These results indicate that 1) the drug-sensitive strain had a competitive growth advantage, causing this strain type to be identified as the sole infecting strain, and that 2) the drug-resistant strain gained the competitive advantage when isoniazid was added and became the predominant strain after treatment. These findings correlate precisely with the patient data (Table) and, we believe, is representative of the in vivo host infection.

**Table:** Details for samples used in a study of the amplification of multidrug-resistant tuberculosis, resulting from competitive growth advantage, during treatment of a drug-sensitive *Mycobacterium tuberculosis* strain in a mixed infection*

Sample	Isolate	Smear	Resistance	MIRU-VNTR
Bronchoalveolar lavage, obtained February 2008	S	+	None	3243323125153242244235-1
Sputum, obtained April 2008	S	++	None	3243323125153242244235-1
Sputum, obtained June 2008	R	+++	INH, ETB	-2434233251533445-443330
Co-cultures 1) broth culture + INH, 2) colonies from broth culture − INH on plate with INH, 3) colonies from broth culture + INH on plates − INH and 4) colonies from broth culture + INH on plates − INH	R	NA	INH	-2434233251533445-443330
Co-cultures 5) broth culture − INH and 6) colonies from broth culture − INH on plates − INH	S	NA	NA	3243323125153242244235-1

*MIRU-VNTR, mycobacterial interspersed repetitive unit–variable number tandem repeat; S, drug sensitive; +, ++, and +++, relative burden of acid-fast bacilli; R, drug resistant; INH, isoniazid; ETB, ethambutol; NA, not applicable.

## Conclusions

We show the selection and subsequent clinically relevant emergence of a drug-resistant *M. tuberculosis* strain after treatment of a drug-sensitive strain in a patient with an initial mixed infection. This case illustrates the prospect of treatment failure for TB caused by mixed infection with strains with different drug susceptibility and growth rates. The proportion of cases of secondary multidrug resistance caused by such initial mixed infections is not known; however, the ability of the resistant strain to outcompete the sensitive strain under treatment and then to potentially transmit further may have substantial implications for the control and prevention of multidrug resistance. 

The case also highlights the urgent need for improved diagnostic techniques that can routinely identify mixed populations of tubercle bacilli. Given the difficulty of detecting TB co-infections by using routine diagnostic microbiology techniques, co-infection is likely underrecognized. Co-infection can currently be ruled out only by using specialized techniques, such as molecular analysis of original sample (pre-culture); analysis of multiple colonies; or the GeneXpert assay (Cepheid, Sunnyvale, CA, USA) (*13*). Rapid detection of mixed infections with distinct drug susceptibility profiles would enable suitably tailored drug regimens from the start of treatment, which could prevent treatment failure and emergence and transmission of drug-resistant strains.
